# Evaluation of the Common Molecular Basis in Alzheimer’s and Parkinson’s Diseases

**DOI:** 10.3390/ijms20153730

**Published:** 2019-07-30

**Authors:** Pratip Rana, Edian F. Franco, Yug Rao, Khajamoinuddin Syed, Debmalya Barh, Vasco Azevedo, Rommel T. J. Ramos, Preetam Ghosh

**Affiliations:** 1 Department of Computer Science, Virginia Commonwealth University, Richmond, VA 23284, USA; 2 Institute of Biological Sciences, Federal University of Para, Belem-PA 66075-110, Brazil; 3 Centre for Genomics and Applied Gene Technology, Institute of Integrative Omics and Applied Biotechnology (IIOAB), Nonakuri, Purba Medinipur, West Bengal 721172, India; 4 Institute of Biological Science, Federal University of Minas Gerais, Belo Horizonte-MG 31270-901, Brazil

**Keywords:** Alzheimer’s disease, Parkinson’s disease, genetics, gene regulatory network, miRNAs

## Abstract

Alzheimer’s disease (AD) and Parkinson’s disease (PD) are the most common neurodegenerative disorders related to aging. Though several risk factors are shared between these two diseases, the exact relationship between them is still unknown. In this paper, we analyzed how these two diseases relate to each other from the genomic, epigenomic, and transcriptomic viewpoints. Using an extensive literature mining, we first accumulated the list of genes from major genome-wide association (GWAS) studies. Based on these GWAS studies, we observed that only one gene (*HLA-DRB5*) was shared between AD and PD. A subsequent literature search identified a few other genes involved in these two diseases, among which SIRT1 seemed to be the most prominent one. While we listed all the miRNAs that have been previously reported for AD and PD separately, we found only 15 different miRNAs that were reported in both diseases. In order to get better insights, we predicted the gene co-expression network for both AD and PD using network analysis algorithms applied to two GEO datasets. The network analysis revealed six clusters of genes related to AD and four clusters of genes related to PD; however, there was very low functional similarity between these clusters, pointing to insignificant similarity between AD and PD even at the level of affected biological processes. Finally, we postulated the putative epigenetic regulator modules that are common to AD and PD.

## 1. Introduction

Alzheimer’s disease (AD) is a complex neurodegenerative disorder, clinically characterized by a gradual decline in memory and impairment of other cognitive functions like communication, movement, higher visual processing, and language inability [1]. About 5.7 million people were living with AD in the U.S. in 2018 [2]. The manifestation of AD is primarily attributed to the extracellular *beta*-amyloid (Aβ42/40) aggregates and intracellular hyperphosphorylated Tau protein that accumulate in the brain of AD patients, causing neuroinflammation and brain cell death. AD is classified into two distinct categories: early onset AD (EOAD), which accounts for less than 5% of the AD population, whereas late-onset AD (LOAD) accounts for about 95% of AD patients [3]. EOAD is a Mendelian pattern disease, whereas LOAD is genetically complex and associated with several genes. The heritability contribution in LOAD is estimated to be around 58–79% [4], and the gene, APOE, has been named as the most important genetic risk factor in LOAD.

On the other hand, Parkinson’s disease (PD) is the second most common age-related neurodegenerative disease. PD is caused by the death of dopamine-generating cells of substantia nigra in the mid-brain region, which affects the function of the central nervous system. Clinically, PD is characterized by syndromes like resting tremor, rigidity, bradykinesia, gait impairment, and postural instability [5,6]. Aggregation of the α-synuclein protein has been considered to be the principal cause for PD.

The relationship between AD and PD is not yet clear. AD and PD share common pathological overlaps despite occurring at different brain locations and having different clinical features. Xie et al. summarized the common pathological overlap between AD and PD, which relates to genes, nicotinic receptors, locus coeruleus, iron, mitochondrial dysfunction, oxidative stress, and neuroinflammation, tau protein, and α-Synuclein protein [7]. Patients with AD have been shown to possess a higher chance of developing PD. One study shows that out of 29 patients with PD, as many as 16 (55%) have mild or severe dementia, which is related to AD [8]. The survival time of PD patients with AD is also lower than that without AD [8]. Both diseases have common risk factors like oxidative stress and aging. Insufficiency of vitamin D has been also reported for both AD and PD patients when compared to the healthy controls [9]. However, so far, no common genetic risk factors have been reported for AD and PD.

In this paper, we examined the genomic, epigenomic, and transcriptomic level similarity between AD and PD. Genome-wide association studies (GWAS) identified more than 50 risk loci associated with LOAD and PD. Furthermore, several studies confirmed the effect of miRNAs in neurodegenerative diseases like AD and PD and reported the associated differentially-expressed microRNAs (miRNAs). miRNAs are non-coding single-stranded RNAs that are very small (20–22 nt) in size and function as negative gene regulators. miRNAs have been also used as a biomarker in early detection and staging information of diseases. Though we know the associated miRNAs and genes in AD and PD, the similarity or possible relationships between them is still unknown. Here, based on a literature search, we identify the different genes and miRNAs that have been associated with AD and PD. Next, we discuss how they may be related in these diseases considering their high likelihood of co-occurrence and predicted common epigenetic modules shared by AD and PD. Lastly, we report the transcriptomic level similarity between AD and PD using regulatory co-expression network prediction and network-based analysis.

## 2. Results

### 2.1. Genetic Associations of AD According to GWAS

EOAD is a Mendelian pattern disease. Three genes, APP, PSEN1, and PSEN2, are considered to be genomic biomarkers in EOAD [10]. These three genes are involved in APP breakdown and Aβ generation. For example, PSEN1 encodes the subunit of γ-secretase, and mutations in PSEN1 is a common cause of EOAD. PSEN1 mutant fibroblasts increase the ratio of Aβ42 to Aβ40 [11]. Mutation in these three genes has been attributed to a wide range (between 12–77%) of EOAD patient [12].

**Figure 1:**
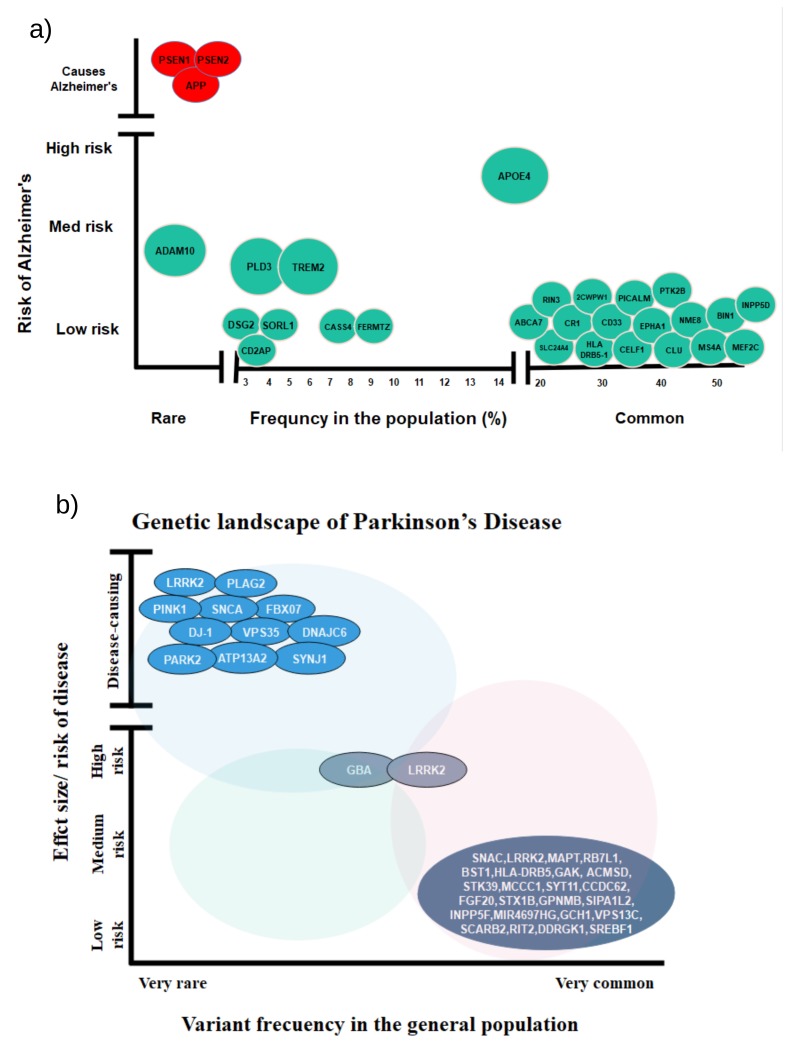
(**a**) Rare and common variants of AD genes and their risk. Red color signifies Mendelian genes, and green signifies non-Mendelian genes (adapted from [15]). (**b**) Rare and common variants of PD genes and their risk (adapted from [16]).

The genetic contribution of EOAD is estimated to be 60–80% [13]. In contrast to EOAD, LOAD is a non-Mendelian disease and demonstrates a complicated relationship with genomics. The first degree relative of an LOAD patient has about a two-times higher probability of developing LOAD in their lifetime than the individual not having first degree LOAD relatives [3]. Genome-wide association studies identified more than 50 risk loci associated with LOAD. A summary of all major GWAS for LOAD is shown in Table 1 and Table 2 and Figure 1. These genes were found to be related to the Aβ pathway, as well as to the immune system, lipid metabolism, and synaptic function. LOAD-related functional effects of these genes are summarized as in [14]:

- Lipid metabolic pathway: APOE, CLU, ABCA7
- Immune system: CLU, CR1, CD33, ABCA7, MS4A, EPHA1
- Complement system: CR1, CLU, ABCA7, CD2AP
- Endocytosis pathway: BIN1, PICLAM, CD2AP

Though genes like PLD3 have higher risk (Figure 1), they are less common in the LOAD population. Therefore, in the following, we briefly review only those genes that are more common.

APOE located on chromosome 19 is the most potent risk factor and the only confirmed susceptibility locus of LOAD. The most common genotype of APOE is APOE3 and has an odds ratio (OR) estimated around 3.2, whereas APOE4, which is present in about 20% of LOAD population, has OR estimated to be around 14.2 [27]. Here, OR is the quantification of the odds that an outcome will occur given a specific exposure [28] compared to the odds of the outcome occurring in the absence of the exposure, with a higher value (>1) reflecting that the exposure is associated with higher odds of outcome and can be designated as a risk factor. However, the APOE2 allele shows some protective effects in AD. APOE has several implications in the AD pathway [29]; it controls lipoprotein metabolism and also affects Aβ clearance by binding with Aβ protein. There is a strong connection of APOE with inflammation, cholesterol transport, and the central nervous system [30]. Neuroimaging studies showed that an APOE4-positive individual has higher deposits of Aβ plaques in the brain compared to an APOE4-negative individual [31]. Few APOE receptors, notably Lrp1, Apoer2, and Vldlr, were identified in the postsynaptic density, which interacts with the synaptic system. Reelin signaling by these receptors activates some pathways that protect Aβ polymerization [32].

Association of the gene CLU (also known as APOJ) and AD has been confirmed in several GWAS experiments. CLU encodes the major brain apolipoprotein, and CLU expression was reported to increase in LOAD brain and also was associated with the reduction of white matter and lower fractional anisotropy in a young, healthy human [33]. This gene is also related to both Aβ clearance and Aβ aggregation. CLU has an essential relationship with inflammation and the immune system [10]. Studies found an increase in CLU concentration in the brain, plasma, and CSF of the patient with AD [34]. Moreover, CLU variants can alter the coupling between the prefrontal cortex and hippocampus [35].

BIN1 is another critical risk locus of LOAD, and altered expression of BIN1 was found in the AD brain. BIN1 mainly increases the risk of AD by modulating tau pathology [36]. Lower BIN1-amphiphysin 2 expression promotes the propagation of tau pathology [37]. BIN1 can also interact with cytoplasmic linker protein CLIP-170; studies found an interaction between tau protein and BIN1 in human neuroblastoma cell [38]. BIN1 is also related to clathrin-mediated endocytosis, which can significantly affect APP processing and Aβ production. A relation between the clathrin-mediated endocytosis gene and toxic effects of Aβ was shown in a study [39]. It also plays a vital role in inflammation. BIN1 participates in phagocytosis and binds to α integrins, which is related to immune response [40]. Studies also found a possible link between the reduction of intracellular Ca++ release and BIN1 protein. Ca++ increase is linked with presenilin mutation, amyloid plaques, and ApoE4 expression, and maintaining calcium homeostasis is essential for normal neuronal function and synaptic transmission [27,41].

Complement receptor 1 (CR1) is the receptor of the C3b/C4b peptide. It encodes monomeric single-pass type I transmembrane glycoprotein, which is involved in immune complement cascade. Four CR1 SNPs (rs646817, rs1746659, rs11803956, and rs12034383) were found to increase Aβ42 concentration in AD patients, which is suggestive of CR1’s role in Aβ metabolism. This gene also might increase Aβ oligomerization over Aβ fibrillogenesis, which causes more neurodegeneration [42]. Further studies suggested that CR1 (rs6656401) is associated with cerebral amyloid angiopathy and vascular amyloid deposition [43]. CR1 mRNA level also correlates with neurofibrillary tangles and phosphorylated tau [42]. CR1 can modulate the complement activation system, which leads to inflammation. A detailed review of this process can be found in [44].

TREM2 is another high-risk gene linked to AD, although it is present in a lower percentage of the population. Studies found the mutation in TREM2 is related to an autosomal recessive form of dementia [45]. Rare missense mutations raise LOAD risk with a similar effect size of APOE [46]. TREM2 R47H raises AD risk by 1.7–3.4-fold [29,47]. TREM2 also correlated with an increase in tau levels in cerebrospinal fluid [48].

### 2.2. Genetic Associations of PD According to GWAS

Genome-wide association studies confirmed that PD has a significant genetic contribution. Previous studies reported about 20 loci and 15 genes related to PD. A summary of all major GWAS for PD is shown in Table 3 and Table 4. From a genetics viewpoint, common variation of loci α-synuclein (SNCA), leucine-rich repeat kinase 2 (LRRK2), and microtubule-associated protein tau (MAPT) showed significant relationships with PD. Moreover, mutation in nine genes, namely SNCA, LRRK2, VPS35, EIF4G1, CHCHD2, PRKN, DJ1, PINK1, and ATP13A2, is associated with the monogenic form of PD [49].

Missense and multiplication mutations in the SNCA gene are believed to be the primary cause of the monogenic form of PD. However, these mutations only account for 10% of PD cases [55]. Mutation in SNCA was first identified in PD in 1997, and until now, five different point mutations have been confirmed as the cause of PD [56]. The non-coding intron in the SNCA gene increases PD susceptibility. Mutated alleles of SNCA change the expression and property of α-synuclein protein, which leads to abnormal aggregation of α-synuclein. The first identified mutation of SNCA was p.A53T, which causes PD. These patients have early age onset (38–49 years) within the Mediterranean origin and rapid disease progression. However, this mutation only accounts for 0.5% of familial and sporadic cases of PD. The second SNCA mutation is p.A30P, with a variable age onset (54–76 years). Cognitive impairment is frequent and early in the patients having this mutation. The third mutation was identified as the heterozygous p.E46K mutation with age ranging from 49–67 years. The fourth mutation p.H50Q was identified in 2013 in a PD patient of age 60 and also in the PD brain-driven DNA. The fifth missense mutation of SNCA is p.G51D; it has an early age onset in the 30s. This mutation leads to PD with unusual clinical and biochemical features. Multiplication of the SNCA gene is more common than these single-point mutations. SNCA duplication and triplication has been reported worldwide. A two-fold expression level of α-synuclein protein has been identified in those patients. SNCA duplication is more common than triplication and has late age onset and slow disease progression compared to the triplication. A common variant of SNCA was also identified as a risk factor of sporadic PD [57].

In 2004, mutation of the LRRK2 gene was identified as a genetic cause of PD. The frequency of LRRK2 mutation in hereditary PD has been estimated to be 4% with an average age onset of 60 years, and sporadic PD is estimated to be around 1% [58]. The most frequent mutation of LRRK2 is G2019S, whereas some of the other common mutations are R1441G, R1441C, Y1699C, and R1441H.

Another monogenic cause of PD is D620N mutation in the VPS35 gene, which was first identified in 2011 in an Austrian family [59]. This mutation accounts for about 1% of familial PD cases. This mutation has a mean age of onset around 53 years with slow disease progression. Other monogenic causes of PD such as the mutation of PARK, PINK1, ATP13A2, and DJ-1, typically have a lower age of onset (<45 years) [49].

Another important gene related to PD is MAPT, which encodes the tau protein. Tau aggregates frequently can be seen in the brain of AD patients. The toxic interaction between tau and α-synuclein may lead to the deposition of both proteins in the brain [60]. α-synuclein also binds with tau, which can reduce the rate of axonal transport. MAPT haplotypes, especially H1 haplotypes, have been identified as a risk factor of PD [61]. MAPT exhibits a mutual regulation with the lysosome function. Interestingly, the autophagy-lysosome pathway is also related to PD [62].

### 2.3. Common Regulator Genes in AD/PD

In order to identify the common regulator genes for AD and PD, we first performed an inner merge of the GWAS reported gene loci for AD and PD. We have found only a single common gene HLA-DRB5 reported for both diseases. HLA-DRB5 has a strong involvement with the immune system. The biological processes related to HLA-DRB5 are adaptive immune response, the T cell receptor signaling pathway, the interferon-gamma-mediated signaling pathway, and antigen processing [63]. Its association with AD and PD has been reported in several other reports [64,65,66].

Outside of GWAS studies, various other studies reported common risk loci for AD and PD, one such gene being SIRT1. It defends against microglia-dependent amyloid β though the NF-kB signaling pathway [67]. Pharmacological and overexpression studies revealed the role of SIRT1 in impacting Aβ plaques [68,69]. A study found that overexpression of SIRT1 suppresses the α synuclein aggregate formation in PD [70], while inactivation of SIRT1 also elevates mitochondrial apoptosis and immune system alterations [71]. Mitochondria are implicated in regulation of cellular redox potency, which is important for normal physiological processes, the deregulation of which is associated with the pathogenesis of aging, neurodegenerative diseases, such as Parkinson’s and Alzheimer’s disease (PD, AD), cardiovascular diseases, inflammation, and metabolic disorders [72]. Additionally, miRNA-34a, miRNA 122, and miRNA 132 inhibit Sirt1; regulation of miRNA-34a and miR132 was reported for AD, while miRNA132 was reported for PD in the literature [73,74,75], which potentially corroborates the involvement of SIRT1 in both AD and PD. A few other genes have also demonstrated shared genetic mechanisms in both AD and PD such as PON1, GSTO, and NEDD9 [7]. PON1 is associated with pesticide metabolism, oxidative stress, and inflammation. A study found that GSTO increases the risk and gene expression level in the brain of both AD and PD patients [76], whereas Li et al. reported NEDD9 as a common risk factor of AD and PD [77]. However, more studies are needed on these genes to determine whether they can be considered as shared risk factors for both diseases.

### 2.4. miRNAs Associated with AD and PD

Large-scale genome annotation reveals that miRNAs play an important role in AD [78]. miRNAs target message transcripts through base pairing, which results in negative gene regulation. Therefore, these miRNAs can alter the expression of critical genes in the AD/PD pathway [79]. The literature reports several miRNAs that have been associated with AD and PD. To identify the role of miRNAs in AD/PD, we performed a systematic review of related miRNAs in AD/PD from the literature, which is shown in Table 5 and Table 6.

**Figure 2:**
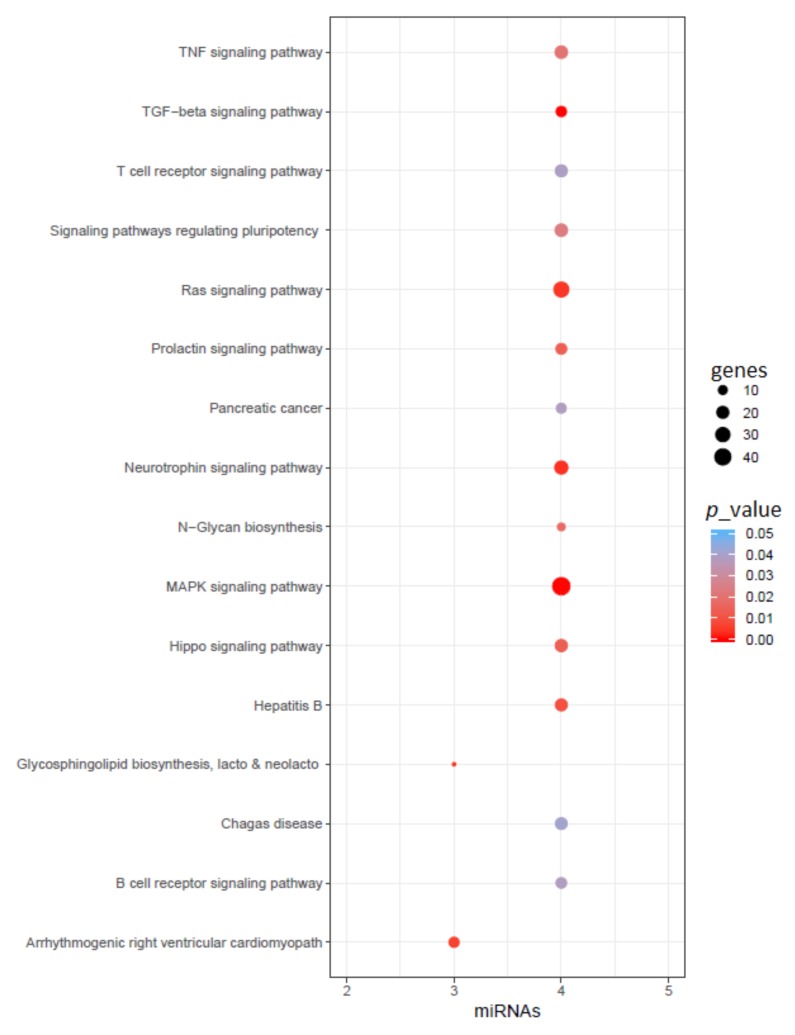
Functional enrichment of reported common miRNAs in AD and PD. Here, color represents the *p*-value of the pathway, size represents common gene targets of the pathway and miRNAs, and the *x*-axis represents the number of related miRNAs in that pathway.

After the literature search, we found a total of 108 miRNAs reported for AD and 91 miRNAs reported for PD. However, only 15 of these miRNAs are common between AD and PD. These miRNAs are hsa-miR-128, hsa-miR-134, hsa-miR-146a, hsa-miR-148b, hsa-miR-151-5p, hsa-miR-16, hsa-miR-181a, hsa-miR-19a, hsa-miR-223, hsa-miR-26a, hsa-miR-29a, hsa-miR-29b, hsa-miR-29c, hsa-miR-30c, and hsa-miR-485-5p. Next, we performed an enrichment analysis of these common miRNA set to identify their function and their target genes. We found a total of 16 KEGG pathways related to these miRNAs (with *p* < 0.05), shown in Figure 2. Some of these pathways (with adjusted *p* < 0.001) are the TGF-beta signaling pathway, MAPK signaling pathway, neurotrophin signaling pathway, glycosphingolipid biosynthesis lacto and neolacto series, Ras signaling pathway, arrhythmogenic right ventricular cardiomyopathy (ARVC), and hepatitis B. Although several of these pathways are not related to the CNS, we have still included them here for completeness.

### 2.5. Putative Epigenetic Regulation Common to AD and PD

**Figure 3:**
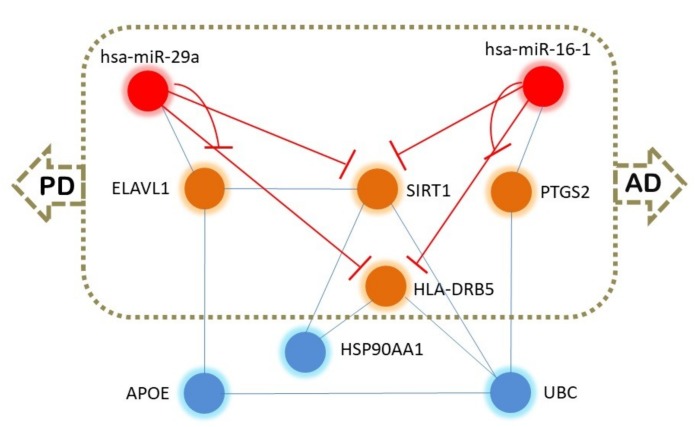
Epigenetic modulation of common molecules in AD and PD. The red color nodes indicate the probable common hub associated with AD and PD pathogenesis.

We analyzed the common 15 miRNAs using the TAM tool (http://www.lirmed.com/tam2/) [97] with the upregulation option, and we observed six miRNAs (hsa-miR-181a, hsa-miR-29a, hsa-miR-29b, hsa-miR-29c, hsa-miR-146a, hsa-miR-148b) associated with Alzheimer’s disease and five miRNAs (hsa-miR-181a, hsa-miR-16, hsa-miR-29a, hsa-miR-29b, hsa-miR-29c) associated with Parkinson’s disease. Therefore, four miRNAs (hsa-miR-181a, hsa-miR-29a, hsa-miR-29b, hsa-miR-29c) are common to both diseases. We used the 15 common miRNAs and the common gene HLA-DRB5 identified from GWAS and analyzed using VisANT 4.0 (http://visant.bu.edu/) [98] for any possible interactions and if there was an intermediate molecule. The analysis revealed that has-miR-29a and has-miR-16 regulate a common pathway associated with AD and PD. hsa-miR-16 interacts with PTGS2 (COX-2, encoded by the gene prostaglandin-endoperoxide synthase 2 (PTGS2)) gene, which is associated with both AD [99,100] and PD [101,102,103,104]. Similarly, the ELAV-like RNA binding protein 1 (ELAV1) interacts with hsa-miR-29a. ELAV1 is associated with AD [105,106], and ELAV1 is found to interact with SIRT1, which is also a marker and target in AD [107,108,109]. UBC is associated with AD [110] and integrates with PTGS2 and HLA-DRB5, which are associated with both AD and PD (Figure 3). Therefore, hsa-miR-29a, hsa-miR-16, ELAVL1, SIRT1, PTGS2, UBC, and HLA-DRB5 may form a hub that could be implicated in providing a common network for AD and PD. DAVID 6.8-based (https://david.ncifcrf.gov) [111] functional analysis revealed that HLA-DRB5, PTGS2, and UBC are associated with Parkinson’s disease and that PTGS2 and SIRT1 are involved in Alzheimer’s disease. Further, ToppGene (https://toppgene.cchmc.org) [112] analysis showed that SIRT1, UBC, HLA-DRB5, MIR29A, and PTGS2 are associated with PD. Therefore, PTGS2 and SIRT1 and their regulatory (immediate or distant) hsa-miR-29a and hsa-miR-16 are probably key molecules common for AD and PD pathogenesis. From these initial results, we tried to explore if the proteins of this hub (ELAVL1, SIRT1, PTGS2, UBC, HLA-DRB5) were also targeted by these two miRNAs (hsa-miR-29a and hsa-miR-16). We used miRWalk (http://mirwalk.umm.uni-heidelberg.de/) [113] and miRDB (http://mirdb.org/) [114], and we observed that SIRT1, ELAVL1, PTGS2, and HLA-DRB5 mRNAs are directly targeted by hsa-miR-16 and hsa-miR-29a/b/c. However, UBC was not found to be targeted by these two miRNAs. To further characterize the epigenetic functionalities of these two miRNAs, we used miRPathDB (https://mpd.bioinf.uni-sb.de) [115]. The miRNA hsa-miR-16 was found to be involved in histone modification, regulation of histone H3-K9 acetylation, positive regulation of histone H3-K9 methylation, positive regulation of histone H3-K4 methylation, regulation of the RNA metabolic process, and rRNA modification in the nucleus and cytosol. The hsa-miR-29 is also involved in pathways associated with histone H3-K4 demethylation, negative regulation of histone H3-K9 methylation, histone ubiquitination, DNA methylation and demethylation, and regulation of the RNA biosynthetic process. Therefore, these two miRNAs may modulate the common epigenetic mechanism behind AD and PD by multiple mechanisms.

### 2.6. Differential Expression Analysis and Functional Enrichment on the GEO Dataset

**Figure 4:**
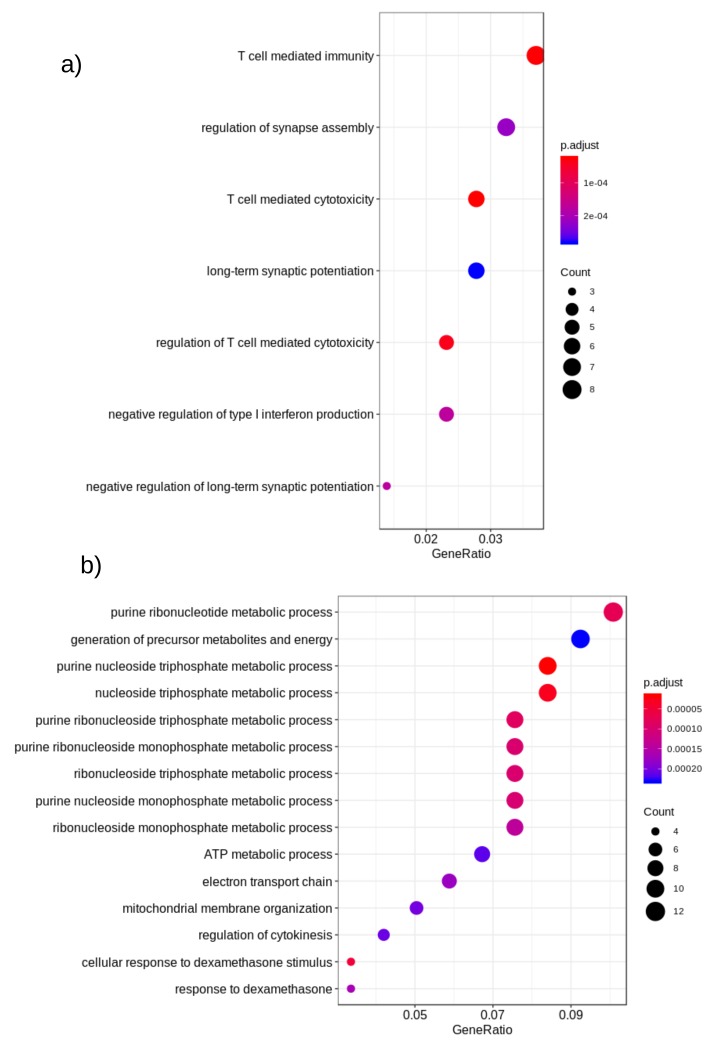
Functional enrichment of differentially-expressed genes of (**a**) AD and (**b**) PD. Here, color denotes the *p*-value of the association and size represents the number of disease-related genes (DE) associated with the pathway. The *x*-axis represents the ratio of the number of disease-related genes (DE) to all related genes to the pathway.

To get better insights into the genomic level similarity between AD and PD, we next used the gene expression data that were downloaded from the Gene Expression Omnibus (GEO) repository [116]. Next, we performed differential expression (DE) analysis on these datasets to identify the important genes in AD/PD. We found that out of the reported gene list, 38 genes were expressed in this dataset in AD, and 1444 genes were expressed in PD (p<0.05), as shown in Table 7. None of these DE genes were however reported in GWAS studies in AD patients. However, in PD, nine DE genes HIP1R, FAM171A1, BIN3, MAPT, RIT2, ALAS1, SH3GL2, ITPKB, and SNCA were reported in PD based on GWAS studies. Next, we performed a functional enrichment analysis on these DE genes. The enriched biological processes are shown in Figure 4.

### 2.7. Gene Co-Expression Network Prediction and Network Analysis on the GEO Dataset

In order to analyze how each gene regulates the others in these diseases, we predicted the gene co-expression network for AD and PD separately using the same GEO dataset. We used only a subset of the genes that were differentially expressed or reported in the GWAS experiments to predict these networks. Next, we took a consensus cutoff of 0.96 for PD and 0.90 for AD to select only the high confidence edges from these networks and visualize the relationship between these genes in AD and PD. AD and PD network data are given in the Supplementary Materials.

**Figure 5:**
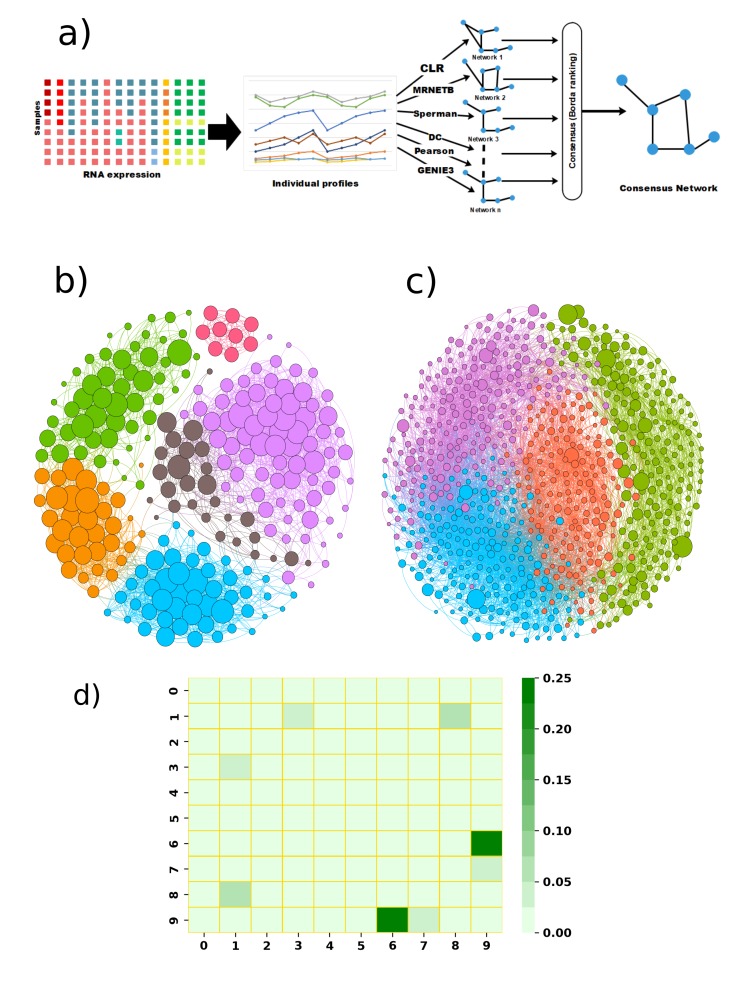
(**a**) Flowchart of gene co-expression network prediction from RNA-seq data; (**b**) gene co-expression network for AD. The six identified clusters are marked with different colors. Node size represents page-rank centrality (larger value means that the gene is more important in that cluster) of each gene. (**c**) Gene co-expression network for PD. The four identified clusters are marked using different colors. (**d**) Functional similarity between identified functionally-enriched clusters between AD and PD. The similarity of clusters was calculated using the Jaccard similarity (see the Methods). Here, Clusters 0–5 are identified as AD clusters, and 6–9 are identified as PD clusters. Darker green color corresponds to higher similarity between clusters.

**Figure 6:**
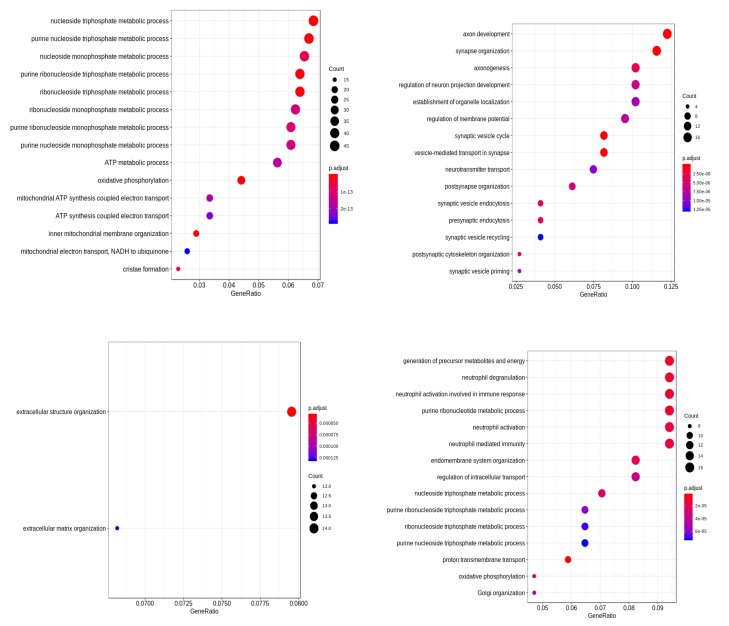
Functional analysis of genes in the different clusters of PD.

**Figure 7:**
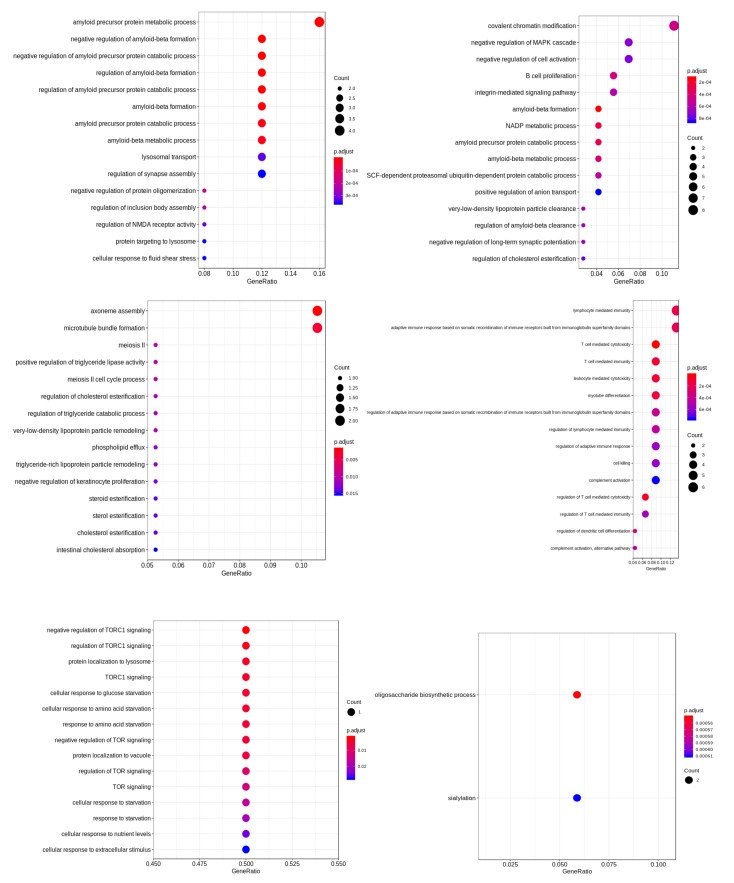
Functional analysis of genes in the different clusters of AD.

Using graph modularity on these networks, we identified a few distinct clusters for AD and PD, which are shown in Figure 5b,c. Each cluster in the network signifies a group of genes that work together closely in the disease. In this dataset, we found six closely-related clusters in AD and four clusters in PD. Next, we functionally enriched the genes in the cluster to relate them to specific biological functions. The identified functions of these clusters are shown in Figure 6 and Figure 7.

Figure 5d visualizes the functional similarity among the clusters of AD and PD. The functional similarity is defined as the number of common functions of the clusters divided by the total number of functions from any two clusters, each chosen from the ones listed in Figure 6 and Figure 7. We found that the functional similarity between the clusters was quite low for AD and PD. This suggests that these clusters affect a different set of functions in each disease.

## 3. Materials and Methods

### 3.1. Literature Mining for GWAS/miRNA Studies

First, we explored the miRNAs/genes reported in different databases like Alzgene [117], PDgene [50], and phenomiR [118] to identify the relevant genes and miRNAs associated with AD and PD. We found that some of these databases are outdated and do not contain current information from the literature. For example, the phenomiR database was last updated in 2011 [118]. Hence, we next manually queried the published literature on or before 2018 through PubMed, ScienceDirect, Scopus, and Google Scholar searches using search terms like “AD/Alzheimer’s + GWAS/gene”, “PD/Parkinson’s + GWAS/gene”, “AD/Alzheimer’s + miRNA/microRNA”, “PD/Parkinson’s + miRNA/microRNA”, “AD/Alzheimer’s + risk loci”, “PD/Parkinson’s + risk loci”, and “LOAD + gene/GWAS/microRNA/miRNAs” to update the information obtained in the previous step for both PD and AD. For GWAS, we only considered the studies having a large number of samples. However, for miRNAs, we listed out all the reported miRNAs in AD/PD as there are fewer reports associated with miRNAs.

### 3.2. Analysis on GEO Data

The transcript expression data for AD/PD were downloaded from the GEO database [119]. For AD, we used GEO accession number GSE84422 as the data source of our studies [120]. GSE84422 contains RNA samples from the brain of 125 human subjects and profiled using Affymetrix Genechip microarrays. For PD, we used GSE accession number GSE20295. It consists of 93 samples taken from different brain regions of PD patients and controls [121].

### 3.3. Gene Coexpression Network Inference Algorithm

Predicting gene–gene interactions is a popular research area and has already been significantly documented in the literature. Genes interact among themselves via transcription factors, through mutual co-expression of a gene group. High-throughput data captured under different conditions by next-generation sequencing (NGS) or RNA-seq make it feasible to computationally predict the gene coexpression network. There are several network inference algorithms that have been implemented over the last few years to infer networks from a snapshot of the transcriptome. However, the performance of these algorithms widely varies over the different datasets and possesses a different inherent bias. There is no single algorithm that performs best in different settings. Hence, in order to predict a high confidence gene coexpression network, we used six popular network inference algorithms. These include two mutual information-based algorithms: (i) context likelihood of relatedness (CLR) [122,123] and (ii) maximum relevance minimum redundancy backward (MRNETB) [124]. We also used basic correlation-based network inference methods: (iii) Pearson and (iv) Spearman correlation, as well as (v) the distance correlation (DC)-based method and (vi) one regression-based gene network inference algorithm called the ensemble of trees (GENIE3) [125]. We next integrated the individual network predictions from each of these six different methods to get one high-confidence interaction network. To integrate the results, we used the wisdom of crowds approach, which is a phenomenon where aggregation of information of a group outperforms the results from an individual. Marbach et al. [126] showed this consensus-based approach outperformed any individual network inference algorithm and predicted a more robust and high-confidence inferred network. Therefore, the wisdom of crowds approach gave us a more accurate picture of gene regulation; this network inference pipeline was previously validated in our prior work [127,128,129]. A flowchart of the steps involved in the gene coexpression network prediction algorithm is shown in Figure 4a.

Unfortunately, some of these network inference algorithms are quite computationally expensive and not feasible to run for thousands of transcripts. Therefore, we re-implemented the parallelized version of these algorithms in CUDA-GPU; the basic idea was to compute the correlation between any gene pair on a different GPU thread. Our implementation achieved about 1000-times speed-up, which enabled us to predict the coexpression network for a large number of transcripts. Predicting high-confidence gene coexpression networks is an essential step towards understanding the role of genes or miRNAs in diseases. It not only shows us how one gene affects another gene in a specific disease, but also gives us the ability to identify how several genes work as a single group in a specific disease.

### 3.4. Gene Set and Functional Similarity Analysis on the GEO Dataset

We used the statistical method LIMMAto find the differentially-expressed (DE) genes from the GEO dataset [130]. Functional analysis on DE genes was performed using the CluterProfiler package in R [131]. We used the Python package networkX and the Gephi tool for analyzing the gene co-expression networks and the subsequent cluster analysis. On the predicted gene coexpression network, we performed modularity-based community detection to identify the clusters in AD/PD. Next, we performed the functional analysis on each cluster to identify the functions of the genes in each cluster. Functional similarity was calculated using the Jaccard index, which is calculated as the common functions between any two clusters divided by the union of functions from the two clusters.

### 3.5. Common miRNA Identification and Pathway Analysis

After identifying causal and common miRNAs between AD and PD, we analyzed the potential effect of these miRNAs in biological pathways. We used the DIANA-miRpath tool to find out the association of critical biological pathways through functional analysis with these deregulated miRNAs [132]. DIANA-miRpath is a bioinformatics tool that identifies experimentally-validated or predicted target genes associated with miRNAs. On the list of genes, it performs merging and meta-analysis algorithms to identify pathways associated with miRNAs. We used the miRTarBasedatabase to predict associated pathways from this tool; miRTarBase predicts biological pathways using only experimentally-confirmed miRNA target genes in a disease [133]. Next, we explored the literature again to gather information about how these miRNAs associate with the identified biological processes in the context of AD and PD.

## 4. Conclusions and Discussions

In this paper, we analyzed the similarity of the two most widely-occurring neurodegenerative diseases: AD and PD. Major GWAS studies identified approximately 50 risk loci for PD and AD. However, we found only one common risk loci (HLA-DRB5) that has been reported for AD and PD in these GWAS studies. HLA-DRB5 has a strong connection with the central nervous system; it has been reported several times before for AD and PD. Other studies from the literature also reported some common risk loci for AD and PD where the gene SIRT1, among others, has been implicated, which plays a dual role in impacting Aβ plaque formation and α-synuclein aggregation. Literature mining also identified 15 common miRNAs that have been reported to be associated with both AD and PD, among which hsa-miR-16 and hsa-miR-29a/b/c could be common epigenetic regulators in these two diseases. The 15 common miRNAs are mainly involved in the TGF-beta signaling pathway, MAPK signaling pathway, neurotrophin signaling pathway, glycosphingolipid biosynthesis, lacto and neolacto series, Ras signaling pathway, and arrhythmogenic right ventricular cardiomyopathy (ARVC).

To get more insights into the reasons behind the co-occurrence of AD and PD, we separately predicted the gene co-expression networks for AD and PD. Using cluster analysis, we found six different clusters in AD and four different clusters in PD, which work together in each of these diseases. We also calculated the functional similarity of these clusters in a combined AD and PD setting, but found very low functional similarity between them; this suggests that very different biological processes are activated in these two diseases, which corroborated our finding that there were not many common genetic loci between AD and PD. Additionally, this may also suggest that the 15 common miRNAs reported for AD and PD may serve as mostly a defense mechanism against brain toxicity and may not play a causal role in either AD or PD.

In a complex heterogeneous disease, different genes’ activation can lead to the same disease outcome [134]. Possibly, AD and PD have different genetic roots, but converge to a similar phenotypic outcome as PD and AD share a few similar symptoms. In this study, we did not considered patient-specific variability of the gene expression while predicting the coexpression networks. One future direction of this study is to consider patient-specific variability to find the genome level similarity between AD and PD.

## Figures and Tables

**Table 1:** Genome-wide association studies (GWAS) in AD.

Study	Ethnic Group	Sample Size	Locus	SNPs
[17]	African-American/ Afro-Caribbean	AD cases: 1009; Control: 6205	CLU PICALM CR1 BIN1 CD2AP EPHA1 MS4A ABCA7	rs2279590 rs3851179 rs6656401 rs744373 rs9349407 rs11767557 rs4938933 rs3865444
[18]	European ancestry, African-American, Japanese, Israeli-Arabic	Stage 1: European ancestry: AD cases: 13,100; control: 13,220 African-American: AD cases: 1472; control: 3511 Japanese: AD cases: 951; control: 894 Israeli Arab: AD cases: 51; control: 64 Stage 2: European ancestry: AD cases: 5813; control: 20,474	PFDN1/HBEGF USP6NL/ECHDC3 BZRAP1-AS1 NFIC	rs1116803 rs7920721 rs2632516 rs9749589
[19]	European	Stage 1: AD cases: 3957; control 9682 Stage 2: AD cases: 2023; control: 2340	TOMM40 PVRL2 APOE CLU PICALM	rs2075650 rs157580 rs6859 rs8106922 rs405509 rs11136000 rs3851179
[20]	European African unspecified NR	European: 16,063 African: 2329 other: 673	TOMM40 APOE PVRL2 APOC1	rs2075650 rs405509 rs8106922 rs6859 rs20769449 rs12721046 rs157582 rs71352238 rs157580 rs439401 rs115881343 rs76366238 rs283815
[21]	Caribbean Hispanic	AD cases: 2451; control: 2063	TOMM40–APOE region FBXL7 CACNA2D	rs394819 rs7500204 Rs743199
[22]	European	AD Cases: 71,880; control 383,378	ADAMTS4 HESX1 CLNK CNTNAP2 ADAM10 APH1B KAT8 ALPK2 AC074212.3	rs4575098 rs184384746 rs114360492 rs442495 rs117618017 rs59735493 rs76726049 rs76320948
[23]	African-Americans	AD cases: 1825; control: 3784	COBL SLC10A2	rs112404845 rs16961023
[24]	African-Americans	AD cases: 1968; control: 3928	ABCA7 HMHA1 GRIN3B	rs115550680 rs115553053 rs115882880 rs145848414

**Table 2:** Genome-wide association studies (GWAS) in AD continued.

Study	Ethnic Group	Sample Size	Locus	SNPs
[25]	European	AD cases: 35,274; control: 59,163	CR1 BIN1 INPP5D HLA-DRB1 TREM2 CD2AP NYAP1g EPHA1 PTK2B CLU SPI1h MS4A2 PICALM SORL1 FERMT2 SLC24A4 ABCA7 APOE CASS4 ECHDC3 ACE MEF2C NME8	rs4844610 rs6733839 rs10933431 rs9271058 rs75932628 rs9473117 rs12539172 rs10808026 rs73223431 rs9331896 rs3740688 rs7933202 rs3851179 rs11218343 rs17125924 rs12881735 rs3752246 rs429358 rs6024870 rs7920721 rs138190086 rs190982 rs4723711
[26]	European	Stage 1: AD cases: 17,008; control: 37,154 Stage 2: AD cases: 8572; Control: 11,312	CR1 BIN1 CD2AP EPHA1 CLU MS4A6A PICALM ABCA7 CD33 HLA-DRB5– HLA-DRB1 PTK2B SORL1 SLC24A4- RIN3 DSG2 INPP5D MEF2C NME8 ZCWPW1 CELF1 FERMT2	rs6656401 rs6733839 rs10948363 rs11771145 rs9331896 rs983392 rs10792832 rs4147929 rs3865444 rs9271192 rs28834970 rs11218343 rs10498633 rs8093731 rs35349669 rs190982 rs2718058 rs1476679 rs10838725 rs17125944 rs7274581

**Table 3:** Genome-wide association studies (GWAS) in PD.

Study	Ethnic Group	Sample Size	Locus	SNPs
[50]	European	PD cases: 5353; control: 5551	GBA-SYT11 RAB7L1-NUCKS1 SIPA1L2 ACMSD-TMEM163 STK39 DLG2 TMEM175-GAK-DGKQ BST1 FAM47E-SCARB2 SNCA HLA-DQB1 GPNMB INPP5F DLG2 MIR4697 LRRK2 CCDC62 GCH1 TMEM229B BCKDK-STX1B MAPT RIT2 DDRGK1 FGF20 MMP16 ITGA8	rs35749011 rs823118 rs10797576 rs6430538 rs1474055 rs12637471 rs34311866 rs11724635 rs6812193 rs356182 rs9275326 rs199347 rs117896735 rs329648 rs76904798 rs11060180 rs11158026 rs2414739 rs14235 rs17649553 rs12456492 rs8118008 rs591323 rs11868035
[51]	Asian	PD cases: 5125; control: 17,604	MCCC1 LRRK2 SNCA DLG2	rs8180209 rs2270968 rs1384236 Rs7479949
[52]	Asian	PD cases: 2011; control: 18,381	PARK16 BST1 SNCA LRRK2	rs16856139 rs823128 rs823122 rs947211 rs823156 rs708730 rs11240572 rs11931532 rs12645693 rs4698412 rs4538475 rs11931074 rs3857059 rs894278 rs6532194 rs1994090 rs7304279 rs4768212 rs2708453 rs2046932

**Table 4:** Genome-wide association studies (GWAS) in PD continued.

Study	Ethnic Group	Sample Size	Locus	SNPs
[53]	European	PD cases: 5333; control: 12,019	SYT11 ACMSD STK39 MCCC1/LAMP3 GAK BST1 SNCA HLA-DRB5 LRRK2 CCDC62/HIP1R MAPT	chr1:154105678 rs6710823 rs2102808 rs11711441 chr4:911311 rs11724635 rs356219 chr6:3258820 rs1491942 rs12817488 rs2942168
[54]	European	PD cases: 6476; control: 302,042	ITPKB IL1R2 SCN3A SATB1 NCKIPSD,CDC71 ALAS1,TLR9, DNAH1,BAP1, PHF7,NISCH, STAB1ITIH3, ITIH4 ANK2, CAMK2D ELOVL7 ELOVL7 ZNF184 CTSB SORBS3, PDLIM2, C8orf58,BIN3 SH3GL2 FAM171A1 GALC COQ7 TOX3 ATP6V0A1, PSMC3IP,TUBG2	rs4653767 rs34043159 rs353116 rs4073221 rs143918452 rs78738012 rs2694528 rs9468199 rs2740594 rs2280104 rs13294100 rs10906923 rs8005172 rs11343 rs4784227 rs601999

**Table 5:** Micro-RNA studies in AD.

Studies	Sample	No. of Patients	No. of Controls	Differential Expression miRNAs
[80]	Plasma	31	37	let-7d-5p, -7g-5p miR-15b-5p, -142-3p, -191-5p,-301a-3p,-545-3p
[81]	Whole Blood	105	150	miR-9, -29a, -29b, -101, -125b, -181c
[82]	Primary hippocampal neuron	NA	NA	miR-9, -181c, -30c, -148b, -20b let-7i
[83]	Brain tissues of the frontal cortex	7	14	miR-29a, -29b,-338-3p
[73]	Human postmortem brain specimens	NA	NA	let-7b, -7c, -7d,-7i, miR-103, -124a, -125a, -125b, -132, -134, -181a, -26a, -26b, -27a, -27b,-29a -29c, -204, -30a-5p, -7, -9
[84]	Serum	208	205	novel miR-36 miR-98-5p, -885-5p, -485-5p,-483-3p,-342-3p, -3158-3p,-30e-5p, -27a-3p, -26b-3p, -191-5p, -151b, let-7g-5p,-7d-5p
[85]	Serum and plasma	32	26	miR-26b-3p, -125b -223, -23a
[74]	Brain tissue postmortem	6	4	miR-338-3p, -219-2-3p, -20a,-17, -106a, -19a, -584, -338-5p, -219-5p, -32, -34c-5p, -16, -151-5p, -181a, -181b, -485-3p, -129-5p, -143, -34a, -124, -149,-136, -138, -145, -129-3p, -381,-128, -432, -378, -29b
[86]	Brain tissue	18	6	miR-9, -125b, -132, -146a, -18
[87]	Serum	19 121	9 86	hmiR-26a-5p, -181c-3p, 126-5p, -22-3p, 148b-5p, -106b-3p, -6119-5p, -1246, -660-5p
[88]	Whole blood	172	109	miR-9-5p, -106a-5p, -106b-5p, -107

**Table 6:** Micro-RNA studies in PD.

Studies	Sample	No. of Patients	No. of Controls	Differential expression miRNAs
[89]	Brain	11	6	miR-34b, miR-34c
[90]	Whole blood	19	13	miR-335.-374a, -199a-3p, -199b-3p, -126, -151-3p, -199a-5p, -151-5p, -29b, -147, -28-5p, -30b, -374b, -19b, -30c, -29c, -301a, -26a
[75]	Cerebrospinal fluid Serum	67	78	miR-132-5p, 19a-3p, -485-5p, -127-3p, -128, -409-3p, -433 -370, -431-3p, -873-3p, -121-3p, -10a, -1224-5p, -4448. miR-388-3p, -16-2-3p, -1294 -30e-3p, -30a-3p
[91]	Frontal cortex	29	33	miR-10b-5p
[92]	Serum	138	112	miR-29c,-146a, -214, and -22
[93]	Whole blood	50	25	miR-24, -30c, -148b, -223, -324-3p
[94]	Serum	10 65	10 65	miR-29c, -19b, -92a, -16, -100 -21, 29a, -451, -19a, -181a, -484 -134, -532-5p, -223
[95]	Cerebrospinal fluid	47	27	miR-1,-103a, -22, -29, -30b, -19-2,-26a, -331-5p, -153, -374 -132-5p, -119a, -485-5p, -127-3p, -151, -28, -301a, -873-3p, -136-3p -19b-3p, 10a-5p, -29c, let-7g-3p
[96]	Cerebrospinal fluid	40	40	miR-27a3p, -125a-5p,-151a-3p, -423-5p let-7f-5p

**Table 7:** Entrez ID of DE genes in AD and PD.

AD DE Gene	PD DE Gene (Top 50 by*p*-Value)
55076, 66005, 114801, 6474, 51084 114041, 2694, 1184, 10859, 347735 53836, 3339, 254295, 51147, 147808 26050, 152573, 51412, 100289341, 27309 285194, 51678, 374920, 135228, 5788 5819, 1051, 4985, 50717, 1293, 100128927 4199, 6921, 2036, 1769, 148066, 57633 10369	4719, 7443, 22877, 5725, 5451 10644, 138151, 100272216, 60496, 7414 2872, 54839, 23313, 4345, 8140 404672, 55750, 10097, 81853, 5521 9201, 55209, 8905, 4190, 902 8382, 56675, 5955, 5567, 7260 5862, 11179, 30827, 400, 23242 37, 51382, 9554, 54541, 9804 801, 29887, 4839, 7994, 64175 23158, 1114, 1353, 65055, 23462

## Supplementary Materials

Supplementary materials can be found at https://www.mdpi.com/1422-0067/20/15/3730/s1.

## Abbreviations

The following abbreviations are used in this manuscript:

AD	Alzheimer’s disease
PD	Parkinson’s disease
GWAS	Genome-wide association
LOAD	Late-onset Alzheimer’s disease
miRNAs	microRNAs
EOAD	Early-onset Alzheimer’s disease
SNP	Single-nucleotide polymorphism
SNCA	Loci α-synuclein
LRRK2	Leucine-rich repeat kinase 2
MAPT	Microtubule-associated protein tau
ARVC	Arrhythmogenic right ventricular cardiomyopathy
DE	Differential expression
GEO	Gene Expression Omnibus
CLR	Context likelihood of relatedness
MRNETB	Maximum relevance minimum redundancy backward
GENIE3	GEne Network Inference with Ensemble of trees
LIMMA	Linear Models for Microarray Data
DC	Distance correlation
GPU	Graphics processing unit
GRN	Gene regulatory network
